# A Case Report of Basal Cell Carcinoma in a Non-Sun-Exposed Area: A Rare Presentation Mimicking Recurrent Perianal Abscess

**DOI:** 10.1155/2018/9021289

**Published:** 2018-11-21

**Authors:** Audrey V. Carr, Edward Feller, Fouad R. Zakka, Rogers C. Griffith, Steven Schechter

**Affiliations:** ^1^Warren Alpert Medical School of Brown University, Providence, RI, USA; ^2^Department of Pathology and Laboratory Medicine, Rhode Island Hospital and Lifespan Medical Center, Warren Alpert Medical School of Brown University, Providence, RI, USA; ^3^Department of Surgery, Warren Alpert Medical School of Brown University, Providence, RI, USA

## Abstract

Basal cell carcinoma (BCC), a common malignancy, arises most often in sun-exposed areas but does rarely occur in non-sun-exposed sites. Prior tissue injury, especially sharp trauma and chronic inflammation, increases the risk of BCC. We describe a 66-year-old male patient with recurrent perianal abscesses who was found to have a large pigmented basal cell carcinoma. The mass was excised without recurrence at two-year follow-up. Perianal BCC is commonly larger at the time of diagnosis than tumors in sun-exposed sites, likely related to delay in diagnosis. Increased size can lead to increased surgical complexity and more pronounced effects on nearby structures. Early detection is important for optimal patient outcomes. In selected patients presenting with a perianal mass, basal cell carcinoma should be included on the differential diagnosis.

## 1. Introduction

Basal cell carcinoma (BCC) is the most common cutaneous malignancy and accounts for approximately 80% of all nonmelanoma skin cancers [1]. It is estimated that 1 in 5 Americans will have a basal cell carcinoma in their lifetime. Incidence increases with age, sun exposure, and male gender [2]. These tumors typically arise in sun-exposed areas; rarely, they occur in nonexposed areas and have been found on the trunk, genitals, nails, axilla, nipple, or sole of the foot [3, 4]. Rarely, these tumors can occur in the perianal region. Three series including 81 cases of perianal BCC have been reported [5–7], and less than 15 individual case reports have been described in the literature [8–17]. Perianal BCCs are noted to be larger in size than those found in more typical, sun-exposed locations [8, 18].

Once recognized, prognosis is generally good. Treatment with surgical excision is typically curative. Although these cancers rarely metastasize, basal cell carcinoma can invade nearby structures. Therefore, early recognition is critical to optimize outcomes. Here, we describe a case of basal cell carcinoma arising in a non-sun-exposed area to alert clinicians to consider BCC in the differential diagnosis when encountering soft tissue perianal masses.

## 2. Case Presentation

**Figure 1:**
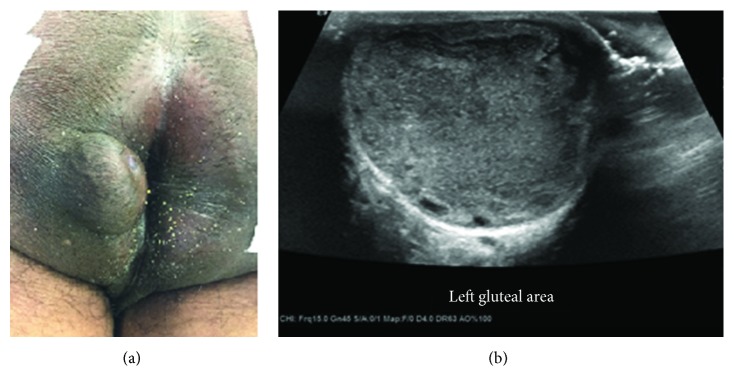
(a) Appearance of the mass at initial presentation. (b) Ultrasound revealed a hyperechoic, well-circumscribed mass.

A 66-year-old male presented with recurrent perianal abscesses over a 12-month period. There was a history of perianal trauma from sitting on broken glass in childhood. Past medical history included vascular dementia, hypertension, hyperlipidemia, atrial fibrillation, and gout. He had no known history of perianal warts, sexually transmitted disease, immune deficiency, inflammatory dermatoses, or arsenic exposure. The patient was a former smoker and had no known family history of malignancy. His primary care physician referred him for ultrasound fine needle aspiration of the abscess, which yielded 15 cc of purulent material (Figure 1). Gram stain and culture were negative for organisms or bacterial growth. Cytology showed atypical squamous cells. Postdrainage differential diagnosis included squamous cell carcinoma, cyst, condyloma, or large abscess.

**Figure 2:**
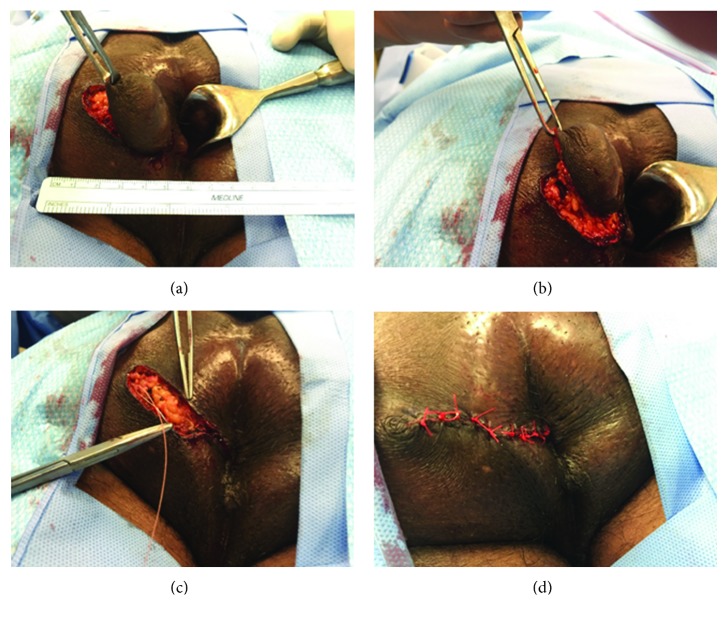
(a, b) Mass measured at 4 cm; the medial margin borders of the gluteal cleft. (c, d) The defect is undermined and closed primarily.

Because of the presence of atypical squamous cells on cytologic analysis, he was referred to a colorectal surgeon; for unclear reasons, the appointment was delayed for 2 months during which time the mass increased in size, prompting concern for a fistula. Rectal examination again revealed a fluctuant mass in the left lateral quadrant. No fistulas were noted on external exam. The abscess was drained surgically yielding purulent fluid with improvement in the patient's pain. The culture did not have any microbial growth. A follow-up exam under anesthesia less than one month later revealed an external sinus tract into the mass but no clear fistula to the anal canal. It was decided to excise the mass completely and close the defect primarily (Figure 2).

**Figure 3:**
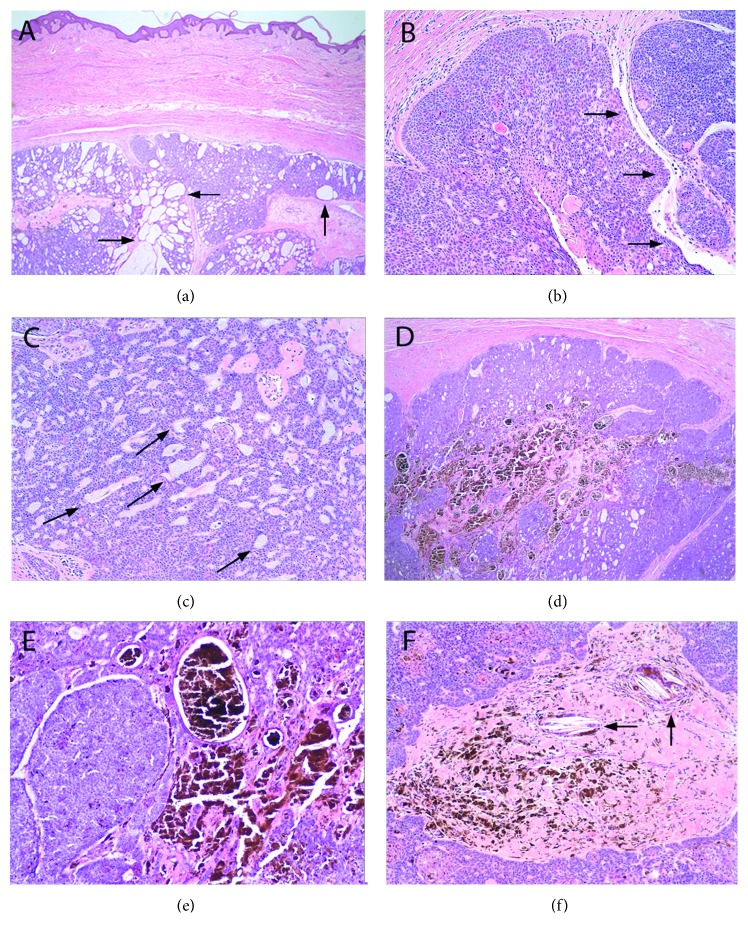
(a) A nodular, well-circumscribed basophilic tumor involved the dermis and displayed areas of cystic architecture (arrows). (b) The tumor cells were basaloid, small, hyperchromatic, and mostly uniform. Nodules exhibited peripheral palisading cells and retraction artifact (arrows). (c) Islands of mucin (arrows) were present within the tumor. (d) Some tumor nodules exhibited dark-brown pigment centrally. (e) The pigment was coarse and acellular. (f) Desmoplastic stroma harboring cholesterol clefts (arrows) and clumps of pigment was seen in nodule centers. (a–f) Hematoxylin and eosin: (a) 20x, (b) 40x, (c) 100x, (d) 40x, (e) 200x, and (f) 100x.

Grossly, the specimen consisted of polypoid skin which contained a well-circumscribed tan-grey nodule measuring 3.0 cm in greatest dimension with a central, folded cystic lumen. Microscopically, a nodular well-circumscribed tumor was present in the dermis (Figure 3(a)) and displayed peripheral palisading cells, desmoplastic changes, and retraction artifact. Tumor cells were small, mostly uniform in shape, and hyperchromatic (Figure 3(b)). The tumor produced mucin which was seen as aggregates within the nodules (Figure 3(c)). Foci of dark-brown acellular pigment consisting of coarse clumped granules were found in the lesion (Figures 3(d) and 3(e)), and some tumor nodules displayed a dense fibrous stroma containing pigment clumps and cholesterol clefts.

Immunohistochemical studies demonstrated tumor cell positivity for cytokeratin 7 (CK7), high molecular weight keratin (CK903), and Ber-EP4. Patchy or focal positivity was observed with cytokeratin CAM 5.2 and epithelial membrane antigen (EMA). Focal melan-A-positive cells were thought to represent benign melanocytes that colonized the tumor. The tumor was negative for melanoma antigen (HMB45) and microphthalmia transcription factor (MITF). The surgical margins were more than 1 mm away from the nearest tumor border.

**Figure 4:**
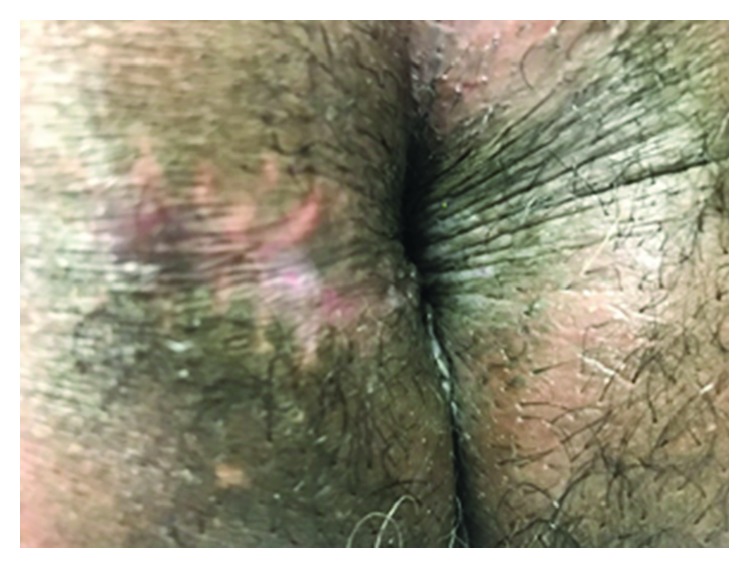
Healed incision site at 10 months postoperatively.

At one-month follow-up, the wound had completely healed. No further adjuvant therapy was performed except periodic surveillance to evaluate for recurrence, as well as dermatologic surveillance to assess for basal cell carcinomas elsewhere. At two-year follow-up evaluation, there had been no recurrence or complications (Figure 4).

## 3. Discussion

Basal cell carcinoma can have diverse histological features, including the presence of pigment and mucin, as seen in this case [19]. Pigmented BCC is a rare clinical and pathological variant which comprises about 6% of total BCCs [20]. Fewer than 100 cases of BCC on the perianal and genital skin have been reported, mainly as individual case reports or small case series [5–17]. In a review of 51 patients, the most common anatomic site was close to the anal orifice, although some reported cases arose from or extended into the anal canal or buttocks as in our patient [5]. The natural history of perianal BCC does not appear to differ from BCC in other locations, although they are typically larger than BCC in other sites, which may be due to delay in diagnosis. When basal cell tumors were originally documented in the perianal area, they were thought to be more aggressive than BCC in other locations; however, this has not been seen in later case reports [6].

Areas of chronic irritation and areas of scar are more likely to develop both squamous cell carcinoma (SCC) and BCC than other healthy tissues [18, 21]. A review of over 1000 cases of carcinomas arising in scar tissue found that 71% of cases were SCC and 12% were BCC [21]. The mean latency from injury to BCC malignancy was 19 years (standard deviation: 22 years). There have also been case reports of carcinoma arising in perianal fistulas, although the majority were rectal adenocarcinomas and the remaining were SCC [22]. It is theorized that scar tissue may lack typical lymphatic channels and therefore these areas do not receive routine surveillance by immune cells, making aberrant cells less likely to be recognized [21]. Human papilloma virus does not appear to be involved in BCC pathogenesis, unlike well-studied associations seen with SCC [5, 23].

Delay in diagnosis is common and likely due to multiple factors, including (1) patient delay in presentation for what they might consider trivial irritation; (2) misdiagnosis of BCC for inflammatory, allergic, or infectious skin lesions; (3) rarity of BCC is sun-protected areas; (4) diversity of macroscopic appearance, ranging from erythematous papules and patches to nodules, plaques, and ulcers, mimicking other more common perianal lesions in the differential diagnoses of a perianal mass (Table 1); and (5) in dark-skinned people, BCC may be pigmented and mistaken for malignant melanoma.

At diagnosis, a total body skin exam should be performed to evaluate for BCC in other locations. Treatment is wide local excision. Tumors arising in areas of prior injury are likely to require complex surgical excision. One analysis found that patients with BCC arising in areas of prior trauma were more likely to require 5 or more stages of Mohs surgery when compared to those BCC related to sun exposure [18]. This difference may be related to increased tissue complexity in already damaged tissue, larger size, or diagnostic delay in some patients. In as many as 95% of cases, local excision is curative and local recurrence is uncommon [5, 6]. Two case reports also reported successful treatment with radiotherapy for patients considered to be poor surgical candidates [10, 12]. Long-term follow-up includes monitoring for BCC in other sites, as patients who have had one BCC may have as much as a 50% risk of developing a second primary BCC within 5 years [24]. However, it is not known if this risk is the same for patients with trauma-related BCC as those with tumors in sun-exposed areas.

Caution should be exercised when assessing prior literature. The bulk of the published experience with perianal BCC consists of case reports or small case series, prone to publication bias favoring unusual, atypical cases that may not reflect actual clinical practice or contemporary reporting and follow-up.

Clinicians must be aware that any chronic buttock lesion, whether arising *de novo* or due to radiation, hidradenitis suppurativa, burns, and trauma (such as sharp injury from glass, as in our patient) can be associated with indolent chronic inflammation and be a precursor to BCC.

## Figures and Tables

**Table 1:** Differential diagnosis of a perianal mass.

Acrochordon (skin tag)	Kaposi's sarcoma
Basal cell carcinoma	Malignant melanoma
Condylomata acuminata	Perianal abscess/fistula
Crohn's disease	Pilonidal cyst/abscess
Cutaneous tuberculosis	Squamous cell carcinoma/Bowen's disease
Epidermoid cyst	Thrombosed external hemorrhoid
Hidradenitis suppurativa	Perianal basaloid (cloacogenic) squamous cell carcinoma
